# Assessment of Drugs Toxicity and Associated Biomarker Genes Using Hierarchical Clustering

**DOI:** 10.3390/medicina55080451

**Published:** 2019-08-08

**Authors:** Mohammad Nazmol Hasan, Masuma Binte Malek, Anjuman Ara Begum, Moizur Rahman, Md. Nurul Haque Mollah

**Affiliations:** 1 Department of Statistics, Bangabandhu Sheikh Mujibur Rahman Agricultural University, Gazipur 1706, Bangladesh; 2 Department of Statistics, Bangladesh Bank, Dhaka 1000, Bangladesh; 3 Bioinformatics Lab., Department of Statistics, University of Rajshahi, Rajshahi 6205, Bangladesh; 4 Animal Husbandry and Veterinary Science, University of Rajshahi, Rajshahi 6205, Bangladesh

**Keywords:** biomarker gene, doses of drugs, fold change gene expression, error rate, toxicity, hierarchical clustering

## Abstract

*Background and objectives:* Assessment of drugs toxicity and associated biomarker genes is one of the most important tasks in the pre-clinical phase of drug development pipeline as well as in toxicogenomic studies. There are few statistical methods for the assessment of doses of drugs (DDs) toxicity and their associated biomarker genes. However, these methods consume more time for computation of the model parameters using the EM (expectation-maximization) based iterative approaches. To overcome this problem, in this paper, an attempt is made to propose an alternative approach based on hierarchical clustering (HC) for the same purpose. *Methods and materials:* There are several types of HC approaches whose performance depends on different similarity/distance measures. Therefore, we explored suitable combinations of distance measures and HC methods based on Japanese Toxicogenomics Project (TGP) datasets for better clustering/co-clustering between DDs and genes as well as to detect toxic DDs and their associated biomarker genes. *Results:* We observed that Word’s HC method with each of Euclidean, Manhattan, and Minkowski distance measures produces better clustering/co-clustering results. For an example, in the case of the glutathione metabolism pathway (GMP) dataset LOC100359539/Rrm2, Gpx6, RGD1562107, Gstm4, Gstm3, G6pd, Gsta5, Gclc, Mgst2, Gsr, Gpx2, Gclm, Gstp1, LOC100912604/Srm, Gstm4, Odc1, Gsr, Gss are the biomarker genes and Acetaminophen_Middle, Acetaminophen_High, Methapyrilene_High, Nitrofurazone_High, Nitrofurazone_Middle, Isoniazid_Middle, Isoniazid_High are their regulatory (associated) DDs explored by our proposed co-clustering algorithm based on the distance and HC method combination Euclidean: Word. Similarly, for the peroxisome proliferator-activated receptor signaling pathway (PPAR-SP) dataset Cpt1a, Cyp8b1, Cyp4a3, Ehhadh, Plin5, Plin2, Fabp3, Me1, Fabp5, LOC100910385, Cpt2, Acaa1a, Cyp4a1, LOC100365047, Cpt1a, LOC100365047, Angptl4, Aqp7, Cpt1c, Cpt1b, Me1 are the biomarker genes and Aspirin_Low, Aspirin_Middle, Aspirin_High, Benzbromarone_Middle, Benzbromarone_High, Clofibrate_Middle, Clofibrate_High, WY14643_Low, WY14643_High, WY14643_Middle, Gemfibrozil_Middle, Gemfibrozil_High are their regulatory DDs. *Conclusions:* Overall, the methods proposed in this article, co-cluster the genes and DDs as well as detect biomarker genes and their regulatory DDs simultaneously consuming less time compared to other mentioned methods. The results produced by the proposed methods have been validated by the available literature and functional annotation.

## 1. Introduction

Assessment of groups of similar toxic doses of drugs (DDs) and their regulatory biomarker genes is the most important objective of toxicity investigation in the pre-clinical phase of drug development process as well as in toxicogenomic studies. Biomarker genes are a set of genes that are differentially expressed in the treatment group of animal compared to the control group. This set of genes is also efficient to differentiate the toxic DDs from the non-toxic DDs. Biomarker genes and their regulatory DDs can be assessed by toxicogenomic study which emerges from toxicology. Toxicology is a field of science which studies the adverse effects of chemicals and environmental exposures in living organisms [1]. The prime objective of this study is the empirical and contextual characterization of adverse effects of chemicals/drugs from tissue, the cell, and the intracellular molecular systems of organisms. Presently, the rapid accumulation of omics (genomics, transcriptomics, proteomics, metabolomics) data, development of sophisticated statistical tools and gene and protein annotation techniques have capitalized the application of gene expression analysis to understand the toxicity mechanism of drugs or chemical compounds and environmental stressors on biological systems. The development of these technologies leads to the development of the new field “toxicogenomics” from toxicology targeting to study the response of the whole genome to DDs or environmental stressors [2,3,4,5,6,7]. The adverse effects of the toxicants in an organism cause pathological changes in certain organs which can be detected by changes in the expression of genes, protein synthesis, and metabolism. Among these, the gene expression or abundant of mRNA is the most sensitive measure of these changes. Thus, toxicogenomics, which enables us to comprehensively analyze gene expression changes caused by an external stimulus in a specific organ, is considered to be one of the most powerful strategies [8,9]. But the toxicogenomic experiment produces a gigantic size of gene expression data. Analysis of this gigantic size of data is very complex and sometimes produces non-robust results for knowledge discovery about biomarker genes and toxicants. Therefore, pathway or molecular network-based gene expression data analysis increases the predictive power and produces more stable biomarkers [10,11,12,13,14].

On the other hand, toxicogenomic data analysis as well as knowledge discovery about the biomarkers and the toxicity of the DDs and environmental stressors often becomes tardy due to the following reasons. (1) Improper selection of statistical/computational tools. (2) Traditional ways of interpretation on the results of computational tools which do not cover the objectives of the study. For example, t-test and Mann–Whitney *U* test [12,15], and ANOVA [16,17] have been used to detect toxicogenomic biomarker genes. However, none of these methods can assess the similar toxic DDs and their associated biomarker genes which is one of the important objectives of toxicity investigation of drug candidates in the pre-clinical phase of the drug development pipeline. The limitation of the above mention methods can be overcome by using hidden variable models [14,18,19]. The hidden variable models are capable to detect toxic DDs and their regulatory biomarker genes by co-clustering DDs and genes. Nevertheless, since hidden variable models are EM (expectation-maximization) [20] based iterative method, these methods require comparatively more time to compute the model parameters. Therefore, to overcome this problem, in this paper, we propose an alternative algorithm based on hierarchical clustering (HC) for co-clustering DDs and genes as well as to discover toxic DDs and their associated biomarker genes. The term cluster analysis refers to the process of assigning data to different groups (clusters) according to their similarity. This approach provides an intuitive method for interpreting complex data such as microarray, transcriptomic, and epigenomic data. There are several types of HC (ward, single, complete, average, mcquitty, median, centroid) approaches whose performance depends on different similarity/distance (euclidean, maximum, manhattan, canberra, minkowski) measures. Every combination of distance and HC methods do not perform equally in grouping objects for all types of datasets. Even the performance of some of these combinations is very poor in some specific fields of study. In the literature, any suitable combination of distance and HC method is not suggested yet for clustering/co-clustering of toxicogenomic data.Hence, in this paper, we explore suitable combinations of distance measures and HC methods based on known Japanese Toxicogenomics Project (TGP) datasets for better clustering/co-clustering between DDs and genes as well as to detect toxic DDs and their associated biomarker genes.

## 2. Methods and Materials

### 2.1. Data Processing

To investigate toxicity of drugs, mRNA abundance in the liver of *Rattus Norvegicus* is measured administering multiple dose levels and time points. A well-designed experiment set to measure gene expression is measured from the treatment group samples where the treatments are the underlying conditions (DDs with time combinations). There are also control samples concurrently to the treatment group samples. The fold change gene expression (FCGE) $y_{pqrt}$ for the $p^{th}\;\left(p=1,\;2,\;\cdots P\right)$ drug, $q^{th}\;\left(q=1,\;2,\;3\right)$ dose level, $t^{th}\;\left(t=1,\;2,\;\cdots ,\;T\right)$ time point, and $r^{th}\;\left(r=1,\;2,\;3\right)$ animal sample can be computed from the gene expression of the treatment and control group of samples using the equation:

(1) $$Y_{pqtr}=log_{2}\left(\frac{x_{pqtr}}{x_{pqtr}^{\prime }}\right)=log_{2}\left(x_{pqtr}\right)-log_{2}\left(x_{pqtr}^{\prime }\right).$$

For single time point this equation can be written as

(2) $$Y_{pqr}=log_{2}\left(\frac{x_{pqr}}{x_{pqr}^{\prime }}\right)=log_{2}\left(x_{pqr}\right)-log_{2}\left(x_{pqr}^{\prime }\right).$$

In the Equation (1) $x_{pqtr}$ is the expression of a gene under the treatment group of animal and $x_{pqtr}^{\prime }$ is the expression of that gene under the control group of animal when the expression is measured at multiple points of time. Similarly, in Equation (2) $x_{pqr}$ and $x_{pqr}^{\prime }$ are the expression of a gene for the treatment and control group of animal, respectively when expression is measured at single time point. The average FCGE value over the animal samples of a gene are ${\bar{Y}}_{pqt.}$ and ${\bar{Y}}_{pq.}$ respectively for multiple and single time point. From these average FCGE values the effect of DDs over the genes can be measured. The values will be positive for upregulated genes and negative for downregulated genes. The datasets of the average FCGE value are the input of our analysis.

### 2.2. Hierarchical Clustering (HC) Algorithms and Distance Measures

The clustering task is solved by the application of various methods depending on the data. Each of these approaches will have peculiarities and the determination of what is the correct or what determines accurate clustering is not easily defined. Hierarchical clustering can proceed using various linkage/clustering and distance methods. The distance method determines how the distance between two observations is calculated. The linkage/clustering method is used when deciding the distance for observations that have already been merged together. Commonly used distance methods are shown in Table 1. In the analysis of biological data, the most commonly used clustering methods are of two types: Hierarchical and non-hierarchical (also known as partitioning). The hierarchical clustering approach builds clusters by repeatedly joining and merging the objects separated by the shortest distance. Following merging of the closest two points the distance matrix is updated and the process repeated until all objects are joined. In this article we have considered five distance methods (euclidean, maximum, manhattan, canberra, minkowski) and seven HC clustering methods (single, complete, average, ward, mcquitty, median, and centroid). We compare all the combinations of distance and HC methods for selecting more suitable combinations for clustering genes or DDs of toxicogenomic data. The description of these HC algorithms is as follows:

#### 2.2.1. Single Linkage

The single linkage HC algorithm clusters objects (genes or doses of chemical compounds) of toxicogenomic data based on the distance or similarity between two pairs of genes/DDs. At the starting, the smallest distance $D=\left\{d_{G_{i},G_{i^{\prime }}}\right\}$ will be found and merge the corresponding genes and form a cluster ($G_{i}G_{i^{\prime }}$). In the next step, the distance between the clusters ($G_{i}G_{i^{\prime }}$) and $G_{i^{\prime \prime }}$ are computed by

(3) $$d_{\left(G_{i},G_{i^{\prime }}\right)G_{i^{\prime \prime }}}=\min\left\{d_{G_{i},G_{i^{\prime \prime }}},d_{G_{i^{\prime }},G_{i^{\prime \prime }}}\right\}$$

to form the cluster ($G_{i},G_{i^{\prime }}G_{i^{\prime \prime }}$). This process continues until all genes merge into a single cluster.

#### 2.2.2. Complete Linkage

In the complete linkage HC algorithm two objects form a cluster together, when their distance is the largest. The general agglomerative algorithm starts finding the minimum entry $D=\left\{d_{G_{i},G_{i^{\prime }}}\right\}$ and merges corresponding genes, such as $G_{i}$ and $G_{i^{\prime }}$, to get cluster ($G_{i},G_{i^{\prime }}$). In the next step clusters ($G_{i}G_{i^{\prime }}$) and $G_{i^{\prime \prime }}$ will be merged into a cluster ($G_{i},G_{i^{\prime }}G_{i^{\prime \prime }}$) based on their maximum distance which is computed as

(4) $$d_{\left(G_{i},G_{i^{\prime }}\right)G_{i^{\prime \prime }}}=\max\left\{d_{G_{i},G_{i^{\prime \prime }}},d_{G_{i^{\prime }},G_{i^{\prime \prime }}}\right\}.$$

This process continues until all genes merge into a single cluster.

#### 2.2.3. Average Linkage

Average linkage treats the distance between two clusters as the average between all pairs of items where one member of a pair belongs to each cluster. We begin searching the distance matrix $D=\left\{d_{G_{i},G_{i^{\prime }}}\right\}$ to find the nearest genes, for example, $G_{i}$ and $G_{i^{\prime }}$ objects are merged to get the cluster ($G_{i}G_{i^{\prime }}$). In the subsequent step, the distance between ($G_{i}G_{i^{\prime }}$) and cluster $G_{i^{\prime \prime }}$ is obtained by

(5) $$d_{\left(G_{i}G_{i^{\prime }}\right)G_{i^{\prime \prime }}}=\frac{\sum_{i}\sum_{i^{\prime \prime }}d_{ii^{\prime \prime }}}{N_{G_{i}G_{i^{\prime }}}N_{G_{i^{\prime \prime }}}}$$

where $d_{ii^{\prime \prime }}$ is the distance between gene i in cluster ($G_{i}G_{i^{\prime }}$) and gene $i^{\prime \prime }$ in cluster $G_{i^{\prime \prime }}$ and $N_{G_{i}G_{i^{\prime }}}$ and $N_{G_{i^{\prime \prime }}}$ are the number of genes in clusters ($G_{i}G_{i^{\prime }}$) and $G_{i^{\prime \prime }}$ respectively.

#### 2.2.4. Centroid

The centroid method involves finding out the mean vector for each of the clusters and talking distance between two centroids. Initially, each of the genes is a cluster then distance between two clusters $G_{i}$ and $G_{i^{\prime }}$ is calculated as:

(6) $$D=d_{\left\{\bar{F\left(G_{i},C_{.}\right)},\;\bar{F\left(G_{i^{\prime }},C_{.}\;\right)}\right\}}$$

#### 2.2.5. Median

The median HC method seeks the median of each of the clusters and measures the distance between two median points. The distance between the median of two clusters $G_{i}$ and $G_{i^{\prime }}$ is

(7) $$D=d_{\left\{F\left(G_{i^{\prime }},C_{Med}\right),\;F\left(G_{i^{\prime }},C_{Med}\right)\right\}}.$$

#### 2.2.6. Ward’s Algorithm

Ward’s HC algorithm clusters objects based on minimizing ‘loss of information’ from joining two groups. This algorithm used error sum of squares (ESS) to measure the loss of information. Firstly, for a given cluster r, let $ESS_{r}$ be the sum of squared deviations of every item in the cluster from the cluster mean (centroid). If there are r clusters, define ESS as $ESS=ESS_{1}+ESS_{2}+\cdots +ESS_{r}$. At each step in the analysis, the union of every possible pair of clusters is considered, and the two clusters whose combination results in the smallest increase in ESS (minimum loss of information) are joined. Initially, each cluster consists of a single item, and, if there are N items, $ESS_{r}=0,\;r=1,\;2,\;\cdots N,$ so ESS=0.

#### 2.2.7. Distance Measures for HC

Most of the distance measure quantifies the distance or dissimilarity among m-dimensional objects or items of a dataset. For example, for a n×m gene-DDs toxicogenomic data matrix consisting of G = $\left(G_{1},\;G_{2},\;\ldots ,\;G_{n}\right)$ genes and C = $\left(C_{1},\;C_{2},\;\ldots ,\;C_{m}\right)$ DDs. We consider the ${\left(i,j\right)}^{th}$ input in the data matrix as $F\left(G_{i},C_{j}\right)$ for convenient using. This input actually represents average FCGE value ${\bar{Y}}_{pq.}$ or ${\bar{Y}}_{pqt.}$ for single or multiple time points. The following are important distance measure used in HC.

### 2.3. Selection of the Suitable Combination of Distance and HC Method

The hierarchical clustering methods group/cluster objects are based on distance matrix which is obtained from the original data matrix. There are also different methods to obtain distance matrix. We investigated the suitability of the combination of distance and HC methods for clustering genes or DDs using DDs clustering error rate (ER) based on known pathway based real datasets. The ER measures the percentage of miss-clustered DDs according to the known DDs which is calculated as:

(8) $$ER=\frac{Miss-clustered\;DDs\;}{Total\;DDs}\times 100.$$

The HC algorithm in combination with distance method which produces the least clustering ER is the more suitable combination of clustering and distance methods for grouping genes or DDs.

### 2.4. Co-Clustering between Genes and DDs and Detection of Toxic DDs and Associated Biomarker Genes Using HC

In the toxicity study, the subsets of DDs regulate the expression profile of the subsets of genes. Accordingly, the genes in a biological pathway perform specific functions and the toxic DDs alter the expression pattern of a subset of biomarker genes in that pathway [19,21]. These biomarker genes and the toxic DDs can be explored from the biomarker co-clusters. For this purpose, more suitable distance and HC methods that produce less ER are used to cluster genes and DDs of toxicogenomic data. Our proposed algorithm follows the following steps to make co-clusters between genes and DDs.

Step 1: Fix the number of clusters in the genes as well as in DDs observing the dendrogram produced by HC according to the researchers’ interest.
Step 2: Take absolute of the FCGE values within intersection areas for all pairs of genes and DDs clusters to give them equal weight in average calculations. Since the FCGE value for upregulated and downregulated genes consists of positive and negative expression values, respectively.
Step 3: Compute the average of the absolute FCGE value for intersection areas of all pairs of genes and DDs clusters.
Step 4: Rank the average FCGE values (computed in step 3) and the respective genes and DDs clusters simultaneously.
Step 5: Assign cluster numbers for genes and DDs newly, based on the ranked average FCGE values which we get from step 4. For example, the gene and DD cluster intersection which produces the largest average FCGE value; we assign both of these gene and DD clusters as cluster 1. Simultaneously, the genes and DDs in cluster 1 together with form co-cluster 1. Similarly, we assign both of the gene and DD cluster as cluster 2 which produces the second largest average FCGE value and they form co-cluster 2 accordingly.

According to the characteristics of toxicogenomic data, a cluster of DDs can form co-cluster with single or more than one cluster of genes, when a DDs cluster might upregulate a set of genes and simultaneously downregulate another set of genes. Researchers consider a gene as differentially expressed or biomarker if its FCGE value is greater than 1.5. In that case, the expression intensity of that gene in the treatment group of samples is almost 3 times larger comparing to its expression in the control group of samples. But when the expression of a gene in the treatment group is 2 times larger than its expression in the control group, the FCGE value of that gene is 1. Therefore, we termed the co-clusters, which average FCGE value greater than one as biomarker co-clusters, and the genes and DDs in these co-clusters as biomarker genes and their regulatory DDs.

### 2.5. Real TGP Datasets to Investigate Clustering Performance

The Japanese Toxicogenomics Project (TGP) [22] collected gene expression data setting out a well-planned experimental condition. There were mainly two types of experiments, one is an in vivo experiment another is an in vitro experiment. The experimental condition pattern of the in vivo experiment was the combination of four time points (3 h, 6 h, 9 h, 24 h) and three dose levels (low, middle, high) and two organs (liver and kidney) of each of the drugs. These treatment conditions were applied on the *Rattus norvegicus* for collecting gene expression data from the target organ. There was also the control animal concurrently for each of the treatment group of animal in the experiment. The FCGE data can be computed from the gene expression data of the treatment group and control group samples produced by this experiment using the Equations (1) and (2). Toxygates a user-friendly interactive data analysis platform as well as database [15] where the FCGE data of the TGP experiment is available. The drugs’ toxicity effects are more clearly visible at 24 h time point compared to the 3 h, 6 h, and 9 h time points [15]. That is why in this paper, we have considered pathway level FCGE data from *Rattus Norvegicus*, in vivo, liver, and single and multiple dose experiments at the 24 h time point. We have downloaded the glutathione metabolism pathway (GMP) and peroxisome proliferator-activated receptor signaling pathway (PPAR-SP) datasets for some selected drugs along with their dose levels whose toxicity mechanism are known [15,23] from Toxygates (http://toxygates.nibiohn.go.jp/toxygates/#columns). Additionally, to investigate the performance of the selected distance and HC methods for clustering toxicogenomic data, datasets for the mentioned pathways for multiple time points and dose levels are also analyzed in this article.

## 3. Results

### 3.1. Selection of Suitable Combination of Distance and HC Methods

As mentioned earlier in the toxicogenomic data, the subsets of DDs regulates the expression patterns of the respective subsets of genes. Therefore, clustering/co-clustering of genes and DDs is an important issue in toxicogenomic studies. HC is a popular and widely used clustering algorithm that uses various distance measures and clustering methods for clustering genes or DDs of toxicogenomic data. However, none of the researchers suggested yet any suitable combination of distance and HC clustering methods for toxicogenomic data. Therefore, to do this we have used two known datasets GMP and PPAR-SP at the 24 h time point [14,15,23] because toxic effects of DDs are more clearly visible at this time point compared to the 3 h, 6 h, or 9 h time points [15]. In the GMP dataset, acetaminophen, methapyrilene, and nitrofurazone are considered as glutathione depleting and erythromycin, hexachlorobenzene, isoniazid, gentamicin, glibenclamide, penicillamine, and perhexilline are considered as non-glutathione depleting drugs [15]. In the PPAR-SP dataset WY-14643, clofibrate, gemfibrozil, benzbromarone, and aspirin are considered as PPARs regulated gene influencing drugs [23] and cisplatin, diltiazem, methapyrilene, phenobarbital, and triazolam are randomly selected drugs. The detail description of the datasets is given in Section 2.5. For comparing the 35 combinations of distance and HC clustering methods, we calculate the ER for both of the datasets in two ways. In the first way, we consider the glutathione depleting and PPARs-regulatory gene influencing drugs in one cluster and the others in another cluster for the respective datasets.

In that case, the FCGE value is merged into a single value averaging over the dose levels (low, middle, and high). In the second way, we consider high and middle doses of glutathione depleting drugs and PPARs-regulated gene influencing drugs in one cluster and other DDs in another cluster for GMP and PPAR-SP datasets, respectively. Therefore, each of the datasets is split into two datasets. For these datasets, the ER is displayed against the 35 combinations of distance and HC clustering methods in Table 2. From this table it is observed that the distance and HC method combinations euclidean: ward, manhattan: ward, and minkowski: ward produce smaller and stable ER in all datasets.

Therefore, we suggest these combinations of distance and HC methods for clustering DDs or genes of toxicogenomic data.

### 3.2. Detection of Biomarker Genes and Their Regulatory DDs from the Co-Clusters

The important objective of the toxicogenomics studies is to explore subset of DDs which have the similar mechanism of action over a subset of genes. This can be done by applying our proposed algorithm described in Section 2.4 on the results obtained from the suitable combination of distance and HC methods. It is observed from the results of the previous Section 3.1, the more suitable combinations of distance and HC methods are euclidean: ward, manhattan: ward, and minkowski: ward. As an example, in this article we show the analysis of GMP and PPAR-SP datasets at 24 h as well as multiple (3 h, 6 h, 9 h, and 24 h) time points using the combinations of HC (ward) and distance (Euclidean) methods.

**Figure 1:**
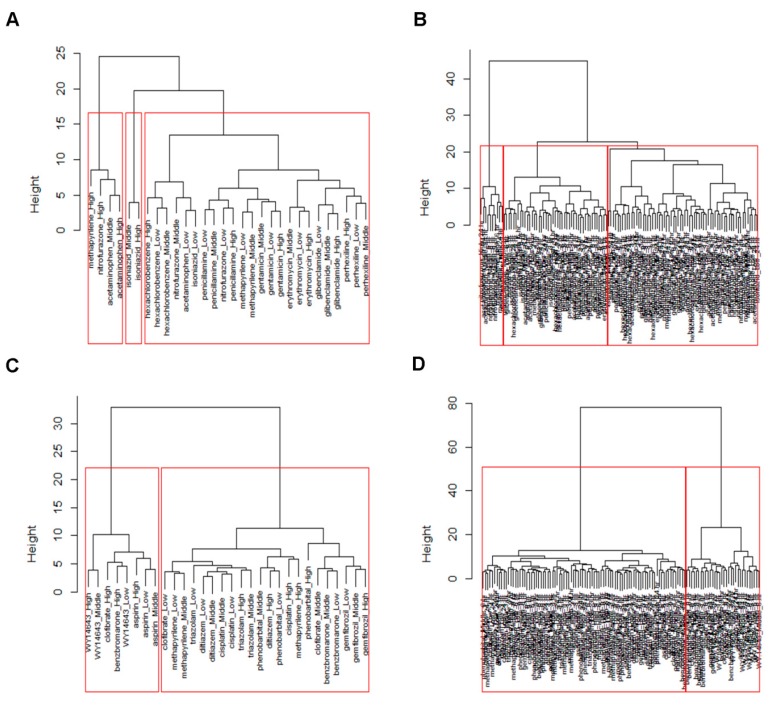
Doses of drugs (DDs) clustering of GMP and PPAR-SP datasets based on the Euclidean distance method in combination with the ward HC method. (**A**) DDs clustering of GMP dataset at 24 h time point. (**B**) DDs clustering of GMP dataset at multiple (3 h, 6 h, 9 h, and 24 h) time points. (**C**) DDs clustering of PPAR-SP dataset at 24 h time point. (**D**) DDs clustering of PPAR-SP dataset at multiple (3 h, 6 h, 9 h, and 24 h) time point.

**Figure 2:**
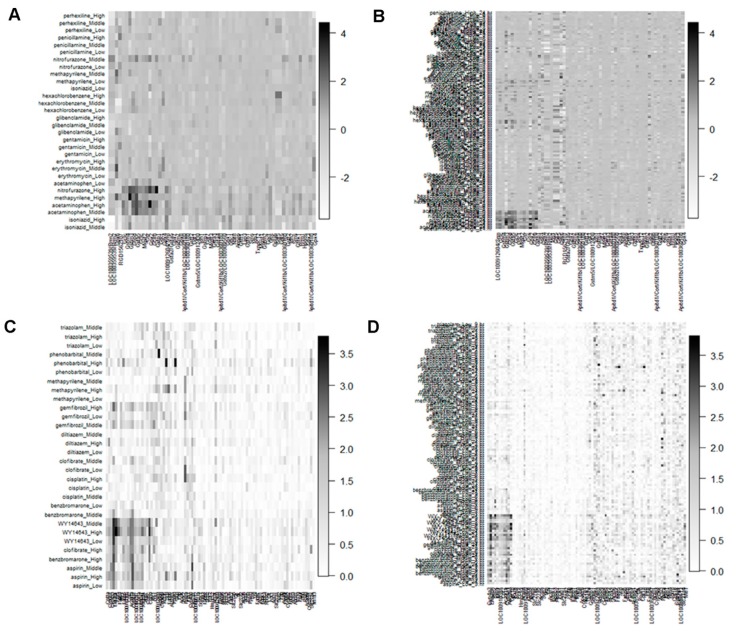
Structural view of co-clusters retrieved by our HC based proposed co-clustering algorithm of the GMP and PPAR-SP datasets. (**A**) GMP dataset for 24 h time point. (**B**) GMP dataset for multiple time points. (**C**) PPAR-SP dataset for 24 h time point. (**D**) PPAR-SP dataset for multiple time points.

The dendrogram of DDs and genes based on the distance (Euclidean) and HC (ward) methods for GMP and PPAR-SP datasets at 24 h as well as multiple time points are depicted in the figures Figure 1 and Figure S1 (Supplementary File), respectively. The ranked clusters/co-clusters (according to average FCGE value within the co-clusters) for the GMP and PPAR-SP datasets are given in Table 3 and Table 4, respectively. In these tables, the genes and DDs cluster numbers within the parenthesis represent the newly assigned cluster numbers based on the proposed co-clustering algorithm described in Section 2.4. For example, in the first row of Table 3 the original HC produced cluster number for both of the gene and DDs is 3. Since, the intersection mean of these genes and DDs cluster is the largest than other genes and DDs cluster intersection mean, we assign the both of the gene and DDs cluster as 1. Figure 2 represents the image of the co-clusters in which genes and DDs are arranged according to the ranked average FCGE values within the co-clusters (Table 3 and Table 4). The biomarker co-clusters along with the proposed method assigned cluster number having the largest average FCGE values (consisting of biomarker genes and their regulatory DDs) are given in Table 5 and Table 6 for GMP and PPAR-SP datasets, respectively. The results generated by the proposed methods for GMP and PPAR-SP datasets are validated by the literature [14,15,23] and functional annotation by the DAVID database [24]. The results of the functional annotation for biomarker genes are given in Table 7, Table 8, Table 9 and Table 10. The detail results of genes and DDs clustering results are given in Supplementary file (Tables S1–S4).

## 4. Discussion

The important objectives of the toxicity investigation in the pre-clinical phase of the drug development process as well as in toxicogenomic studies are the subsets of DDs which have the similar mechanism of action over the respective subsets of genes and to assess toxic DDs and their regulatory toxicogenomic biomarker genes. With a view to satisfy these objectives, different authors have incorporated a number of statistical tools in their works. For example, t-test and Mann–Whitney *U* test [12,15], and ANOVA [16,17] were used for the exploration of biomarker genes. Nonetheless, these methods cannot satisfy the mentioned objectives. Although, there are few statistical methods [14,18,19] for the assessment of doses of drugs (DDs) toxicity and their associated biomarker genes, these methods consume more time for computation of the model parameters using the EM (expectation-maximization) [20] based iterative approaches. To overcome this problem, in this paper, we have proposed an alternative approach based on hierarchical clustering (HC) for the same purpose. However, there are several types of HC approaches whose performance depend on different similarity/distance measures. Therefore, we explored suitable combinations of distance measures and HC methods based on Japanese Toxicogenomics Project (TGP) datasets for better clustering/co-clustering between DDs and genes as well as to detect toxic DDs and their associated biomarker genes.

We investigated the performance of 35 combinations of distance (euclidean, maximum, manhattan, canberra, minkowski) and HC (ward, single, complete, average, mcquitty, median, centroid) methods based on the known real glutathione metabolism pathway (GMP) and PPAR signaling pathway (PPAR-SP) datasets [15,23] using ER. It is observed that the combinations euclidean: ward, manhattan: ward, and minkowski: ward produce more stable and lower ER for the mentioned datasets. Therefore, we have proposed ward’s HC methods in combination with distance methods euclidean, manhattan, or minkowski for clustering/co-clustering genes and DCCs of toxicogenomic data. For example, we have analyzed GMP and PPAR-SP for single and multiple time points datasets using the distance and HC method combination euclidean: ward based proposed co-clustering algorithm described in Section 2.4. In the case of the glutathione metabolism pathway (GMP) dataset LOC100359539/Rrm2, Gpx6, RGD1562107, Gstm4, Gstm3, G6pd, Gsta5, Gclc, Mgst2, Gsr, Gpx2, Gclm, Gstp1, LOC100912604/Srm, Gstm4, Odc1, Gsr, Gss are the biomarker genes explored from biomarker co-clusters (for single and multiple time points datasets combined) and Acetaminophen_Middle, Acetaminophen_High, Methapyrilene_High, Nitrofurazone_High, Nitrofurazone_Middle, Isoniazid_Middle, Isoniazid_High are their regulatory (associated) DDs. Similarly, for the PPAR signaling pathway (PPAR-SP) dataset Cpt1a, Cyp8b1, Cyp4a3, Ehhadh, Plin5, Plin2, Fabp3, Me1, Fabp5, LOC100910385, Cpt2, Acaa1a, Cyp4a1, LOC100365047, Cpt1a, LOC100365047, Angptl4, Aqp7, Cpt1c, Cpt1b, Me1 are the biomarker genes and Aspirin_Low, Aspirin_Middle, Aspirin_High, Benzbromarone_Middle, Benzbromarone_High, Clofibrate_Middle, Clofibrate_High, WY14643_Low, WY14643_High, WY14643_Middle, Gemfibrozil_Middle, Gemfibrozil_High are their regulatory DDs. These results are validated by the available literature [14,15,23] and functional annotation.

## 5. Conclusions

Overall, the study has shown that the proposed methods have significant advantage over the existing biomarker gene detection as well as co-clustering methods due to the following reasons.

- Detect the biomarker genes and the regulatory (associated) DDs simultaneously.
- The method safe time, since it requires less time for preparing results compared to the other EM based iterative co-clustering methods.
- The results produced by the method conform to the literature and database results.

## Figures and Tables

**Table 1:** Important distance measures used in hierarchical clustering.

Distance Measure	Mathematical Form
Euclidean	$d_{G_{i},G_{i^{\prime }}}={\left(\overset{m}{\underset{j=1}{\sum }}{\left(F\left(G_{i},C_{j}\right)-F\left(G_{i^{\prime }},C_{j}\right)\right)}^{2}\right)}^{1/2}$
Minkowski	$d_{G_{i},G_{i^{\prime }}}={\left(\overset{m}{\underset{j=1}{\sum }}{\left|F\left(G_{i},C_{j}\right)-F\left(G_{i^{\prime }},C_{j}\right)\right|}^{v}\right)}^{1/v}$
Manhattan	$d_{G_{i},G_{i^{\prime }}}=\overset{m}{\underset{j=1}{\sum }}\left|F\left(G_{i},C_{j}\right)-F\left(G_{i^{\prime }},C_{j}\right)\right|$
Canbera	$d_{G_{i},G_{i^{\prime }}}=\overset{m}{\underset{j=1}{\sum }}\frac{\left|F\left(G_{i},C_{j}\right)-F\left(G_{i^{\prime }},C_{j}\right)\right|}{F\left(G_{i},C_{j}\right)+F\left(G_{i^{\prime }},C_{j}\right)}$
Maximum	$d_{G_{i},G_{i^{\prime }}}=max_{j}\left|F\left(G_{i},C_{j}\right)-F\left(G_{i^{\prime }},C_{j}\right)\right|$

**Table 2:** Percent of error rate (ER) for 35 combinations of distance and HC clustering methods calculated from the glutathione metabolism and PPAR signaling pathway datasets.

Sl	Combination of Distance and HC Clustering Methods	Drug Clustering ER for GMP Data	Drug Clustering ER for PPAR-SP Data	DDs Clustering ER for GMP Data	DDs Clustering ER for PPAR-SP Data
**1**	**euclidean:ward**	**10**	**0**	**6.666666667**	**20**
2	euclidean:single	10	40	16.66666667	36.66666667
3	euclidean:complete	10	30	26.66666667	20
4	euclidean:average	10	40	26.66666667	20
5	euclidean:mcquitty	40	40	26.66666667	13.33333333
6	euclidean:median	40	40	3.333333333	26.66666667
7	euclidean:centroid	40	40	16.66666667	30
8	maximum:ward	10	0	16.66666667	10
9	maximum:single	10	40	16.66666667	36.66666667
10	maximum:complete	20	0	16.66666667	26.66666667
11	maximum:average	10	40	26.66666667	36.66666667
12	maximum:mcquitty	10	40	26.66666667	36.66666667
13	maximum:median	40	40	26.66666667	36.66666667
14	maximum:centroid	40	40	16.66666667	30
**15**	**manhattan:ward**	**10**	**0**	**6.666666667**	**20**
16	manhattan:single	40	40	16.66666667	36.66666667
17	manhattan:complete	10	30	3.333333333	20
18	manhattan:average	10	40	26.66666667	20
19	manhattan:mcquitty	10	40	3.333333333	20
20	manhattan:median	40	40	26.66666667	36.66666667
21	manhattan:centroid	40	40	16.66666667	30
22	canberra:ward	50	10	30	20
23	canberra:single	50	10	23.33333333	36.66666667
24	canberra:complete	50	10	30	20
25	canberra:average	50	10	30	23.33333333
26	canberra:mcquitty	50	40	30	23.33333333
27	canberra:median	50	40	40	36.66666667
28	canberra:centroid	50	40	33.33333333	36.66666667
**29**	**minkowski:ward**	**10**	**0**	**6.666666667**	**20**
30	minkowski:single	10	40	16.66666667	36.66666667
31	minkowski:complete	10	30	26.66666667	20
32	minkowski:average	10	40	26.66666667	20
33	minkowski:mcquitty	40	40	26.66666667	13.33333333
34	minkowski:median	40	40	3.333333333	26.66666667
35	minkowski:centroid	40	40	16.66666667	30

**Table 3:** Doses of drug and gene co-clustering mean (ranked) of the glutathione metabolism pathway datasets for the combination (Euclidean: ward) of distance and hierarchical clustering methods.

**Euclidean: ward, Dataset: glutathione metabolism pathway at 24 h time point**	
**Gene and compound co-cluster**	**Co-cluster mean**
Gene-Cluster-3(1): Compound-Cluster-3(1)	2.5550390
Gene-Cluster-2(2): Compound-Cluster-2(2)	1.6619841
Gene-Cluster-3(1): Compound-Cluster-2(2)	0.8249199
Gene-Cluster-3(1): Compound-Cluster-1(3)	0.8129127
Gene-Cluster-2(2): Compound-Cluster-3(1)	0.5994644
Gene-Cluster-1(3): Compound-Cluster-3(1)	0.5991663
Gene-Cluster-1(3): Compound-Cluster-2(2)	0.4653372
Gene-Cluster-2(2): Compound-Cluster-1(3)	0.3402437
Gene-Cluster-1(3): Compound-Cluster-1(3)	0.2481545
**Euclidean: ward, Dataset: glutathione metabolism pathway at 3 h, 6 h 9 h, and 24 h time points**	
**Gene and compound co-cluster**	**Co-cluster mean**
Gene-Cluster-3(1): Compound-Cluster-2(1)	1.2954907
Gene-Cluster-1(2): Compound-Cluster-1(2)	0.6118177
Gene-Cluster-2(3): Compound-Cluster-1(2)	0.5850958
Gene-Cluster-3(1): Compound-Cluster-1(2)	0.5157947
Gene-Cluster-3(1): Compound-Cluster-3(3)	0.3513179
Gene-Cluster-1(2): Compound-Cluster-2(1)	0.3360666
Gene-Cluster-2(3): Compound-Cluster-2(1)	0.3285539
Gene-Cluster-1(1): Compound-Cluster-3(3)	0.2478899
Gene-Cluster-2(3): Compound-Cluster-3(3)	0.2424664

**Table 4:** Doses of drug and gene co-clustering mean (ranked) of the PPAR signaling pathway datasets for the combination (Euclidean: ward) of distance and hierarchical clustering methods.

**Euclidean: ward, Dataset: PPAR signaling pathway at 24 h time point**	
**Gene and compound co-cluster**	**Co-cluster mean**
Gene-Cluster-1(1): Compound-Cluster-1(1)	1.5972416
Gene-Cluster-3(2): Compound-Cluster-2(2)	0.6596625
Gene-Cluster-3(2): Compound-Cluster-1(1)	0.6522308
Gene-Cluster-1(1): Compound-Cluster-2(2)	0.4973316
Gene-Cluster-2(3): Compound-Cluster-1(1)	0.3994878
Gene-Cluster-2(3): Compound-Cluster-2(2)	0.2378871
**Euclidean: ward, Dataset: PPAR signaling pathway at 3 h, 6 h 9 h, and 24 h time points**	
**Gene and compound co-cluster**	**Co-cluster mean**
Gene-Cluster-3(1): Compound-Cluster-2(1)	1.5863836
Gene-Cluster-1(2): Compound-Cluster-2(1)	0.5842037
Gene-Cluster-1(2): Compound-Cluster-1(2)	0.4385611
Gene-Cluster-3(1): Compound-Cluster-1(2)	0.4025768
Gene-Cluster-2(3): Compound-Cluster-2(1)	0.2569643
Gene-Cluster-2(3): Compound-Cluster-1(2)	0.1757952

**Table 5:** Biomarker co-clusters consisting of biomarker genes and their regulatory doses of drugs explored by the combination (Euclidean: ward) of distance and hierarchical clustering methods for glutathione metabolism pathway datasets.

Biomarker Genes	Regulatory Doses of Drugs
**Euclidean: ward, Dataset: glutathione metabolism pathway at 24 h time point**	
**Gene-cluster-3:** LOC100359539/Rrm2, LOC100359539/Rrm2, Gpx6, RGD1562107	**DCCs-cluster-3:** isoniazid_Middle, isoniazid_High
**Gene-cluster-2:** Gclc, Gstm4, Gstm3, G6pd, Gsta5, Gclc, Mgst2, Gsr, Gpx2, Gclm, Gstp1	**DCCs-cluster-2:** acetaminophen_Middle, acetaminophen_High, methapyrilene_High, nitrofurazone_High
**Euclidean:ward, Dataset: glutathione metabolism pathway at 3 h, 6 h 9 h, and 24 h time points**	
**Gene-cluster-2:** LOC100912604/Srm, Gclc, Gstm4, Gstm3, G6pd, Gsta5, Gclc, Odc1, Mgst2, Gsr, Gss, Gpx2, Gclm, Gstp1	**DCCs-cluster-3:** acetaminophen_High_24.hr, acetaminophen_Middle_24.hr, methapyrilene_High_6.hr, methapyrilene_High_24.hr, methapyrilene_High_9.hr, nitrofurazone_High_24.hr, nitrofurazone_High_6.hr, nitrofurazone_Middle_6.hr, nitrofurazone_High_9.hr, nitrofurazone_Middle_9.hr,

**Table 6:** Biomarker co-clusters consisting of biomarker genes and their regulatory doses of drugs explored by the combination (Euclidean: ward) of distance and hierarchical clustering methods for PPAR signaling pathway datasets.

Biomarker Genes	Regulatory Doses of Drugs
**Euclidean: ward, Dataset: PPAR signaling pathway at 24 h time point**	
**Gene-cluster-1:** Cpt1a, Cyp8b1, Cyp4a3, Ehhadh, Plin5, Fabp3, Me1, Fabp5, LOC100910385, Cpt2, Acaa1a, Cyp4a1, LOC100365047, Cpt1a, LOC100365047, Angptl4, Aqp7, Cpt1c, Cpt1b, Me1	**DCCs-cluster-1:** aspirin_Low, aspirin_High, aspirin_Middle, benzbromarone_High, clofibrate_High, WY14643_Low, WY14643_High, WY14643_Middle
**Euclidean: ward, Dataset: PPAR signaling pathway at 3 h, 6 h 9 h, and 24 h time points**	
**Gene-cluster-3:** Cyp4a3, Ehhadh, Plin2, Plin5, Me1, LOC100910385, Cpt2, Acaa1a, Cyp4a1, Angptl4, Cpt1b	**DCCs-cluster-2:** aspirin_Low_9.hr, aspirin_Low_24.hr, aspirin_High_9.hr, aspirin_High_24.hr, aspirin_Middle_24.hr, benzbromarone_Middle_6.hr, benzbromarone_High_9.hr, benzbromarone_High_3.hr, benzbromarone_Middle_9.hr, enzbromarone_High_24.hr, benzbromarone_High_6.hr, benzbromarone_Middle_3.hr, clofibrate_Middle_6.hr, clofibrate_High_24.hr, clofibrate_Middle_9.hr, clofibrate_High_6.hr, clofibrate_High_9.hr, gemfibrozil_High_24.hr, gemfibrozil_Middle_24.hr, gemfibrozil_High_9.hr, WY.14643_High_6.hr, WY.14643_Middle_6.hr, WY.14643_Middle_24.hr, WY.14643_Low_3.hr, WY.14643_Low_24.hr, WY.14643_Middle_9.hr, WY.14643_Low_6.hr, WY.14643_High_9.hr, WY.14643_Middle_3.hr, WY.14643_High_3.hr, WY.14643_Low_9.hr, WY.14643_High_24.hr

**Table 7:** Functional annotation of KEGG pathway on the biomarker genes in co-cluster-1 discovered by the distance and HC method combination Euclidean: ward, Dataset: glutathione metabolism pathway at 24 h time point.

Term	Count	%	*p*-Value	FDR	Genes
rno00480: Glutathione metabolism	2	66.66	7.48E−3	2.04E−38	RGD1562107, Gpx6

**Table 8:** Functional annotation of KEGG pathway on the biomarker genes in co-cluster-2 discovered by the distance and HC method combination Euclidean: ward, Dataset: glutathione metabolism pathway at 24 h time point.

Term	Count	%	*p*-Value	Genes
rno00480: Glutathione metabolism	10	100	3.85E−20	Mgst2, Gpx2, G6pd, Gclm, Gsr, Gsta5, Gclc, Gclc, Gstp1, Gstm3, Gstm4
rno00980: Metabolism of xenobiotics by cytochrome P450	5	50.0	7.43E−7	Mgst2, Gsta5, Gstp1, Gstm3, Gstm4
rno00982: Drug metabolism—cytochrome P450	5	50.0	7.87E−7	Mgst2, Gsta5, Gstp1, Gstm3, Gstm4
rno05204: Chemical carcinogenesis	5	50.0	2.14E−6	Mgst2, Gsta5, Gstp1, Gstm3, Gstm4
rno04918: Thyroid hormone synthesis	2	20.0	0.076	Gpx2, Gsr

**Table 9:** Functional annotation of KEGG pathway on the biomarker genes in co-cluster-1 discovered by the distance and HC method combination Euclidean: ward, Dataset: PPAR signaling pathway at 24 h time point.

Term	Count	%	*p*-Value	Genes
rno03320: PPAR signaling pathway	13	76.47	4.88E−24	Cpt1b, Aqp7, Cpt1c, Cpt1a, Cyp4a3, Cpt1a, Cpt2, Cyp8b1, Fabp3, Ehhadh, Acaa1a, Cyp4a1, Angptl4, Fabp5
rno00071: Fatty acid degradation	8	47.06	3.16E−13	Cpt1b, Cpt2, Ehhadh, Acaa1a, Cpt1c, Cpt1a, Cyp4a3, Cpt1a, Cyp4a1
rno01212: Fatty acid metabolism	6	35.29	1.67E−8	Cpt1b, Cpt2, Ehhadh, Acaa1a, Cpt1c, Cpt1a, Cpt1a
rno04920: Adipocytokine signaling pathway	3	17.65	0.0067	Cpt1b, Cpt1c, Cpt1a, Cpt1a
rno04922: Glucagon signaling pathway	3	17.65	0.0117	Cpt1b, Cpt1c, Cpt1a, Cpt1a
rno04152: AMPK signaling pathway	3	17.65	0.0187	Cpt1b, Cpt1c, Cpt1a, Cpt1a
rno01100: Metabolic pathways	6	35.29	0.0500	Me1, Me1, Cyp8b1, Ehhadh, Acaa1a, Cyp4a3, Cyp4a1
rno00280: Valine, leucine and isoleucine degradation	2	11.76	0.0885	Ehhadh, Acaa1a

**Table 10:** Functional annotation of KEGG pathway on the biomarker genes in co-cluster-1 discovered by the distance and HC method combination Euclidean: ward, Dataset: PPAR signaling pathway at 3 h, 6 h, 9 h, and 24 h time points.

Term	Count	%	*p*-Value	Genes
rno03320: PPAR signaling pathway	7	63.63	5.49E−12	Cpt1b, Cpt2, Ehhadh, Acaa1a, Cyp4a3, Angptl4, Cyp4a1
rno00071: Fatty acid degradation	6	54.54	1.37E−10	Cpt1b, Cpt2, Ehhadh, Acaa1a, Cyp4a3, Cyp4a1
rno01212: Fatty acid metabolism	4	36.36	1.09E−5	Cpt1b, Cpt2, Ehhadh, Acaa1a
rno01100: Metabolic pathways	5	45.45	0.0172	Me1, Ehhadh, Acaa1a, Cyp4a3, Cyp4a1
rno00280: Valine, leucine and isoleucine degradation	2	18.18	0.0486	Ehhadh, Acaa1a
rno00590: Arachidonic acid metabolism	2	18.18	0.0709	Cyp4a3, Cyp4a1
rno00830: Retinol metabolism	2	18.18	0.0726	Cyp4a3, Cyp4a1
rno04146: Peroxisome	2	18.18	0.0743	Ehhadh, Acaa1a
rno04750: Inflammatory mediator regulation of TRP channels	2	18.18	0.0994	Cyp4a3, Cyp4a1

## Supplementary Materials

The following are available online at https://www.mdpi.com/1010-660X/55/8/451/s1. Figure S1: Gene clustering of glutathione metabolism (GMP) and PPAR signaling pathway (PPAR-SP) datasets based on Euclidean distance method in combination with ward HC method. Table S1: Gene and DDs clusters as well as co-clusters generated by the proposed co-clustering algorithm based on the combination of distance (Euclidean) and HC (ward) methods for glutathione metabolism pathway datasets at 24 h time point. Table S2: Gene and DDs clusters as well as co-clusters generated by the proposed co-clustering algorithm based on the combination of distance (Euclidean) and HC (ward) methods for glutathione metabolism pathway datasets at 3 h, 6 h, 9 h, and 24 h time points. Table S3: Gene and DDs clusters as well as co-clusters generated by the proposed co-clustering algorithm based on the combination of distance (Euclidean) and HC (ward) methods for PPAR signaling pathway dataset at 24 h time point. Table S4: Gene and DCCs clusters as well as co-clusters generated by the proposed co-clustering algorithm based on the combination of distance (Euclidean) and HC (ward) methods for PPAR signaling pathway dataset at 3 h, 6 h, 9 h, and 24 h time points.
