# Supplementary material for: Assessment of Drugs Toxicity and Associated Biomarker Genes Using Hierarchical Clustering
Source: Medicina (Kaunas). 2019 Aug 8;55(8):451. doi: 10.3390/medicina55080451 (PMC6723056; doi:10.3390/medicina55080451)
Supplement: Supplementary file 1 [file medicina-55-00451-s001.pdf]

Supplementary Material

Assessment of Drugs Toxicity and Associated Biomarker Genes using Hierarchical Clustering

Mohammad Nazmol Hasan[1], Masuma Binte Malek[2], Anjuman Ara Begum[3], Moizur Rahman[4] and Md. Nurul Haque Mollah[3*]

[1] Department of Statistics, Bangabandhu Sheikh Mujibur Rahman Agricultural University, Gazipur-1706, Bangladesh.

[2] Department of Statistics, Bangladesh Bank, Dhaka, Bangladesh.

[3] Bioinformatics Lab., Department of Statistics, University of Rajshahi, Rajshahi-6205, Bangladesh;

[4] Animal Husbandry and Veterinary Science, University of Rajshahi, Rajshahi-6205, Bangladesh;

[*]Correspondence:
Corresponding Author: Md. Nurul Haque Mollah
E-mail: mollah.stat.bio@ru.ac.bd, nazmol.sat.bsmrau@gmail.com

Contents

Supplementary Figures

Figure S1: Gene clustering of glutathione metabolism (GMP) and PPAR signaling pathway (PPAR-SP) datasets based on euclidean distance method in combination with ward HC method.

Supplementary Tables

Table S1: Gene and DDs clusters as well as co-clusters generated by the proposed co-clustering algorithm based on the combination of distance (Euclidean) and HC (ward) methods for glutathione metabolism pathway datasets at 24 hour time point.

Table S2: Gene and DDs clusters as well as co-clusters generated by the the proposed co-clustering algorithm based on the combination of distance (Euclidean) and HC (ward) methods for glutathione metabolism pathway datasets at 3 hour, 6 hour, 9 hour and 24 hour time points.

Table S3: Gene and DDs clusters as well as co-clusters generated by the proposed co-clustering algorithm based on the combination of distance (Euclidean) and HC (ward) methods for PPAR signaling pathway dataset dataset at 24 hour time point.

Table S4: Gene and DCCs clusters as well as co-clusters generated by the proposed co-clustering algorithm based on the combination of distance (Euclidean) and HC (ward) methods for PPAR signaling pathway dataset at 3 hour, 6 hour, 9 hour and 24 hour time points.

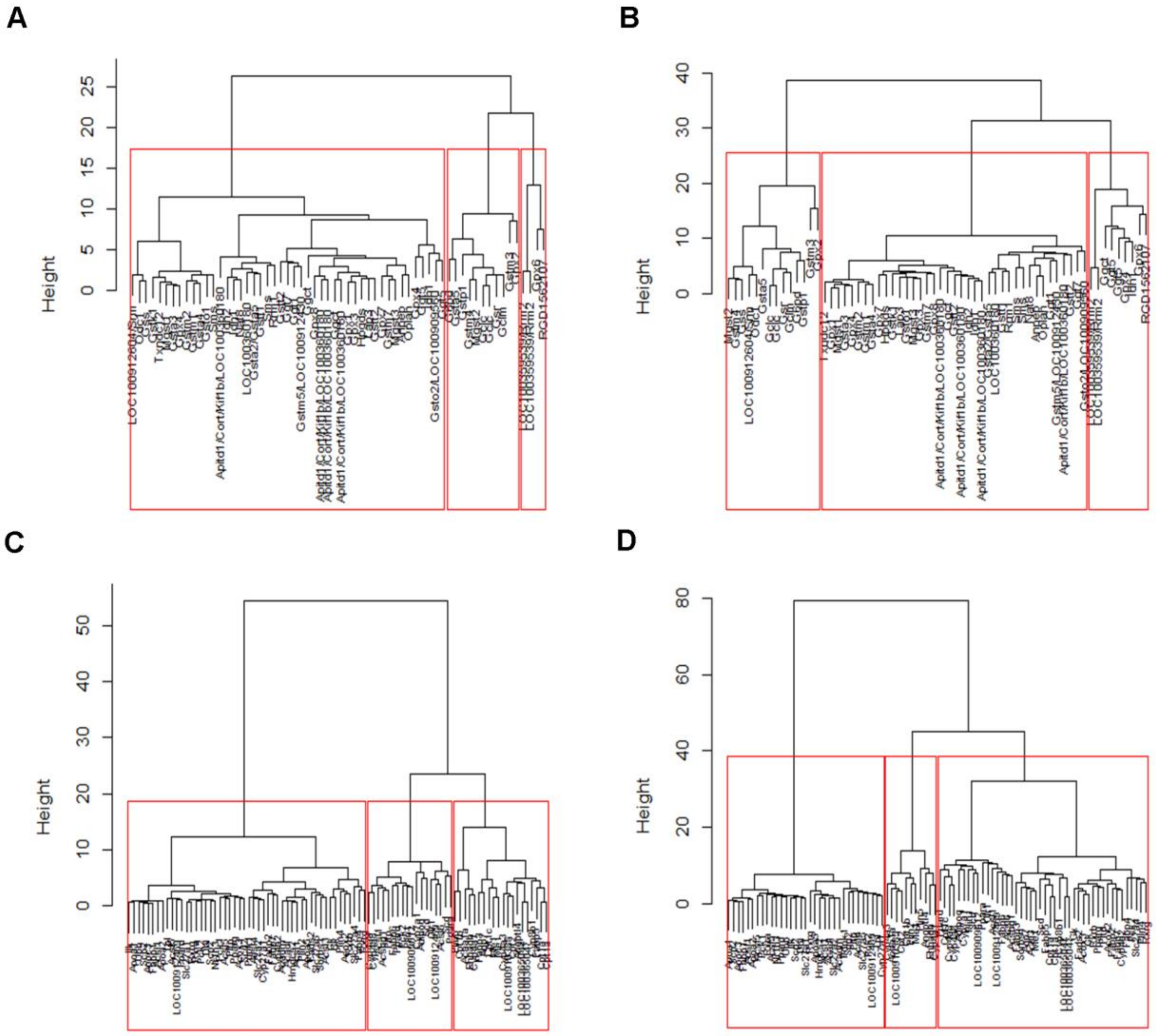

Figure S1: Gene clustering of glutathione metabolism (GMP) and PPAR signaling pathway (PPAR-SP) datasets based on euclidean distance method in combination with ward HC method. **A)** Gene clustering of GMP dataset at 24 hour time point. **B)** Gene clustering of GMP dataset at multiple (3 hour, 6 hour, 9hour and 24 hour) time point. **C)** Gene clustering of PPAR-SP dataset at 24 hour time point. **D)** Gene clustering of PPAR-SP dataset at multiple (3 hour, 6 hour, 9hour and 24 hour) time point.

Table S1: Gene and DDs clusters as well as co-clusters generated by the proposed co-clustering algorithm based on the combination of distance (Euclidean) and HC (ward) methods for glutathione metabolism pathway datasets at 24 hour time point.

**Gene Clusters**		
**Cluster-1**	**Cluster-2**	**Cluster-3**
LOC100359539/Rrm2, LOC100359539/Rrm2, Gpx6, RGD1562107	Gclc, Gstm4, Gstm3, G6pd, Gsta5, Gclc, Mgst2, Gsr, Gpx2, Gclm, Gstp1	Ggct, Gpx4, LOC100912604/Srm, Gsta2/Gsta5, Gpx7, Gstm7, Gpx8, Apitd1/Cort/Kif1b/LOC100360180, LOC100360180, Gstt2, Ggt1, Gstm5/LOC100912430, Idh1, Gstm1, Ggt7, Mgst3, Gsta3, Gsta4, Apitd1/Cort/Kif1b/LOC100360180, Gsto2/LOC100909560, Gpx1, Odc1, Nat8, Anpep, Gstt1, Lap3, Gsto1, Sms, Idh1, Txndc12, Mgst1, Gstm2, Ggct, Gss, Oplah, Ggt5, Ggt5, Apitd1/Cort/Kif1b/LOC100360180, Gstt2, Idh2, Sms, Gstk1, Rrm1, Hpgds, Gpx3, Apitd1/Cort/Kif1b/LOC100360180, Gpx4
**DDs Clusters**		
isoniazid_Middle, isoniazid_High	acetaminophen_Middle, acetaminophen_High, methapyrilene_High, nitrofurazone_High	acetaminophen_Low, erythromycin_Low, erythromycin_Middle, erythromycin_High, gentamicin_Low, gentamicin_Middle, gentamicin_High, glibenclamide_Low, glibenclamide_Middle, glibenclamide_High, hexachlorobenzene_Low, hexachlorobenzene_Middle, hexachlorobenzene_High, isoniazid_Low, methapyrilene_Low, methapyrilene_Middle, nitrofurazone_Low, nitrofurazone_Middle, penicillamine_Low, penicillamine_Middle, penicillamine_High, perhexiline_Low, perhexiline_Middle, perhexiline_High

Table S2: Gene and DDs clusters as well as co-clusters generated by the proposed co-clustering algorithm based on the combination of distance (Euclidean) and HC (ward) methods for glutathione metabolism pathway datasets at 3 hour, 6 hour, 9 hour and 24 hour time points.

Cluster-1	Cluster-2	Cluster-3
**Gene Clusters**		
LOC100912604/Srm, Gclc, Gstm4, Gstm3, G6pd, Gsta5, Gclc, Odc1, Mgst2, Gsr, Gss, Gpx2, Gclm, Gstp1	Ggct, Gpx4, LOC100359539/Rrm2, LOC100359539/Rrm2, Idh1, Ggt5, Ggt5, Gpx6, RGD1562107	Gsta2/Gsta5, Gpx7, Gstm7, Gpx8, Apitd1/Cort/Kif1b/LOC100360180, LOC100360180, Gstt2, Ggt1, Gstm5/LOC100912430, Idh1, Gstm1, Ggt7, Mgst3, Gsta3, Gsta4, Apitd1/Cort/Kif1b/LOC100360180, Gsto2/LOC100909560, Gpx1, Nat8, Anpep, Gstt1, Lap3, Gsto1, Sms, Txndc12, Mgst1, Gstm2, Ggct, Oplah, Apitd1/Cort/Kif1b/LOC100360180, Gstt2, Idh2, Sms, Gstk1, Rrm1, Hpgds, Gpx3, Apitd1/Cort/Kif1b/LOC100360180, Gpx4
**DDs Clusters**		
acetaminophen_High_24.hr,<br>acetaminophen_Middle_24.hr,<br>methapyrilene_High_6.hr,<br>methapyrilene_High_24.hr,<br>methapyrilene_High_9.hr,<br>nitrofurazone_High_24.hr,<br>nitrofurazone_High_6.hr,<br>nitrofurazone_Middle_6.hr,<br>nitrofurazone_High_9.hr,<br>nitrofurazone_Middle_9.hr,	acetaminophen_Middle_6.hr,<br>acetaminophen_Low_3.hr,<br>acetaminophen_Low_6.hr,<br>acetaminophen_High_6.hr,<br>acetaminophen_High_3.hr,<br>hexachlorobenzene_High_24.hr,<br>hexachlorobenzene_Low_3.hr,<br>hexachlorobenzene_High_6.hr,<br>hexachlorobenzene_High_3.hr,<br>methapyrilene_Low_3.hr,<br>erythromycin_Middle_9.hr,<br>erythromycin_Middle_6.hr,<br>erythromycin_High_6.hr,<br>erythromycin_High_9.hr,<br>erythromycin_Low_6.hr,<br>glibenclamide_Low_24.hr,<br>glibenclamide_High_6.hr,<br>glibenclamide_Middle_6.hr,<br>glibenclamide_Low_6.hr,<br>glibenclamide_High_24.hr,<br>glibenclamide_Middle_24.hr,<br>isoniazid_Low_6.hr,<br>isoniazid_Low_3.hr,<br>isoniazid_Middle_6.hr,<br>isoniazid_High_9.hr,<br>isoniazid_Middle_9.hr,<br>isoniazid_High_6.hr,<br>isoniazid_Middle_3.hr,<br>isoniazid_High_3.hr,<br>perhexiline_High_6.hr,<br>perhexiline_Low_6.hr,<br>perhexiline_Middle_6.hr,	acetaminophen_High_9.hr,<br>acetaminophen_Low_24.hr,<br>acetaminophen_Middle_3.hr,<br>acetaminophen_Low_9.hr,<br>acetaminophen_Middle_9.hr,<br>hexachlorobenzene_Middle_3.hr,<br>hexachlorobenzene_Low_9.hr,<br>hexachlorobenzene_Low_24.hr,<br>hexachlorobenzene_Low_6.hr,<br>hexachlorobenzene_Middle_24.hr,<br>hexachlorobenzene_High_9.hr,<br>hexachlorobenzene_Middle_6.hr,<br>hexachlorobenzene_Middle_9.hr,<br>methapyrilene_Middle_24.hr,<br>methapyrilene_Low_9.hr,<br>methapyrilene_Middle_3.hr,<br>methapyrilene_High_3.hr,<br>methapyrilene_Low_6.hr,<br>methapyrilene_Middle_9.hr,<br>methapyrilene_Low_24.hr,<br>methapyrilene_Middle_6.hr,<br>nitrofurazone_Low_3.hr,<br>nitrofurazone_High_3.hr,<br>nitrofurazone_Low_9.hr,<br>nitrofurazone_Low_24.hr,<br>nitrofurazone_Low_6.hr,<br>nitrofurazone_Middle_24.hr,<br>nitrofurazone_Middle_3.hr,<br>erythromycin_Middle_24.hr,<br>erythromycin_High_24.hr,<br>erythromycin_Low_24.hr,<br>erythromycin_High_3.hr,

**Cluster-1**	**Cluster-2**	**Cluster-3**
	gentamicin_High_6.hr,<br>gentamicin_Middle_6.hr,<br>penicillamine_Low_3.hr,<br>penicillamine_High_3.hr,<br>penicillamine_Low_24.hr,<br>penicillamine_Low_6.hr,<br>penicillamine_High_9.hr,<br>penicillamine_Low_9.hr,<br>penicillamine_Middle_3.hr"<br>penicillamine_Middle_9.hr"<br>penicillamine_Middle_24.hr"<br>penicillamine_Middle_6.hr"<br>penicillamine_High_6.hr"	erythromycin_Low_9.hr,<br>erythromycin_Middle_3.hr,<br>erythromycin_Low_3.hr,<br>glibenclamide_High_3.hr,<br>glibenclamide_Low_9.hr,<br>glibenclamide_High_9.hr,<br>glibenclamide_Middle_9.hr,<br>glibenclamide_Low_3.hr,<br>glibenclamide_Middle_3.hr,<br>isoniazid_Low_24.hr,<br>isoniazid_Middle_24.hr,<br>isoniazid_Low_9.hr,<br>isoniazid_High_24.hr,<br>perhexiline_Low_9.hr,<br>perhexiline_Low_24.hr,<br>perhexiline_High_24.hr,<br>perhexiline_Middle_3.hr,<br>perhexiline_High_9.hr,<br>perhexiline_Low_3.hr,<br>perhexiline_High_3.hr,<br>perhexiline_Middle_9.hr,<br>perhexiline_Middle_24.hr,<br>gentamicin_Middle_24.hr,<br>gentamicin_Middle_9.hr,<br>gentamicin_Middle_3.hr,<br>gentamicin_Low_24.hr,<br>gentamicin_High_24.hr,<br>gentamicin_High_9.hr,<br>gentamicin_Low_3.hr,<br>gentamicin_High_3.hr,<br>gentamicin_Low_9.hr,<br>gentamicin_Low_6.hr,<br>penicillamine_High_24.hr,

Table S3: Gene and DDs clusters as well as co-clusters generated by the proposed co-clustering algorithm based on the combination of distance (Euclidean) and HC (ward) methods for PPAR signaling pathway dataset dataset at 24 hour time point.

**Cluster-1**	**Cluster-2**	**Cluster-3**
**Gene Clusters**		
Cpt1a, Cyp8b1, Cyp4a3, Ehhadh, Plin5, Fabp3, Me1, Fabp5, LOC100910385, Cpt2, Acaa1a, Cyp4a1, LOC100365047, Cpt1a, LOC100365047, Angptl4, Aqp7, Cpt1c, Cpt1b, Me1	Olr1, LOC100912469, Adipoq, Cyp4a8, Acsl6, Scd, Fabp6, Acsbg1, Ppard, Scd, Pltp, Acsl1, Pck2, Ppara, Plin1, Cyp4a8, Cyp7a1, LOC100909612	Rxra, Sorbs1, Plin2, Fabp2, Scp2, Pck2, Hmgcs2, Acsl4, LOC100912469, Ppard, Apoa5, Acox2, Acox3, Acsl5, Fads2, Slc27a5, Gk, Ilk, Slc27a2, Acsl5, Scp2, Rxrb, Pparg, Dbi, Lpl, Fabp7, Acsl1, Pdpk1, Apoc3, Apoa2, Gk, Pck1, Acsl1, Slc27a1, Scd2, Pltp, Acadl, Fabp4, Cyp4a2, Fabp1, Acsl3, Apoa1, Rxrg, Plin4, Ubb, Rxrg, Acadm, Cyp27a1, Slc27a4, Nr1h3
**DDs Clusters**		
aspirin_Low, aspirin_High, aspirin_Middle, benzbromarone_High, clofibrate_High, WY14643_Low, WY14643_High WY14643_Middle	benzbromarone_Middle, benzbromarone_Low, cisplatin_Middle, cisplatin_Low, cisplatin_High, clofibrate_Low, clofibrate_Middle, diltiazem_Low, diltiazem_High, diltiazem_Middle, gemfibrozil_Middle, gemfibrozil_Low, gemfibrozil_High, methapyrilene_Low, methapyrilene_High, methapyrilene_Middle, phenobarbital_Low, phenobarbital_High, phenobarbital_Middle, triazolam_Low, triazolam_High, triazolam_Middle,	

Table S4: Gene and DCCs clusters as well as co-clusters generated by the proposed co-clustering algorithm based on the combination of distance (Euclidean) and HC (ward) methods for PPAR signaling pathway dataset at 3 hour, 6 hour, 9 hour and 24 hour time points.

Cluster-1	Cluster-2	Cluster-3
**Gene Clusters**		
Cyp4a3, Ehhadh, Plin2, Plin5, Me1, LOC100910385, Cpt2, Acaa1a, Cyp4a1, Angptl4, Cpt1b	Cpt1a, Cyp8b1, Sorbs1, Olr1, LOC100912469, Adipoq, Fabp2, Cyp4a8, Fabp3, Acox2, Acox3, Acsl6, Fads2, Fabp5, Scd, Gk, Fabp6, Rxrb, Pparg, Fabp7, Acsbg1, LOC100365047, Gk, Ppard, Cpt1a, Scd, Pltp, Fabp4, LOC100365047, Acsl1, Cyp4a2, Pck2, Acsl3, Ppara, Plin1, Aqp7, Plin4, Rxrg, Cpt1c, Cyp4a8, Cyp7a1, LOC100909612, Slc27a4, Me1	Rxra, Scp2, Pck2, Hmgcs2, Acsl4, LOC100912469, Ppard, Apoa5, Acsl5, Slc27a5, Ilk, Slc27a2, Acsl5, Scp2, Dbi, Lpl, Acsl1, Pdpk1, Apoc3, Apoa2, Pck1, Acsl1, Slc27a1, Scd2, Pltp, Acadl, Fabp1, Apoa1, Rxrg, Ubb, Acadm, Cyp27a1, Nr1h3
**DDs Clusters**		
aspirin_Low_9.hr,<br>aspirin_Low_24.hr,<br>aspirin_High_9.hr,<br>aspirin_High_24.hr,<br>aspirin_Middle_24.hr,<br>benzbromarone_Middle_6.hr,<br>benzbromarone_High_9.hr,<br>benzbromarone_High_3.hr,<br>benzbromarone_Middle_9.hr,<br>benzbromarone_High_24.hr,<br>benzbromarone_High_6.hr,<br>benzbromarone_Middle_3.hr,<br>clofibrate_Middle_6.hr,<br>clofibrate_High_24.hr,<br>clofibrate_Middle_9.hr,<br>clofibrate_High_6.hr,<br>clofibrate_High_9.hr,<br>gemfibrozil_High_24.hr,<br>gemfibrozil_Middle_24.hr,<br>gemfibrozil_High_9.hr,<br>WY.14643_High_6.hr,<br>WY.14643_Middle_6.hr,<br>WY.14643_Middle_24.hr,<br>WY.14643_Low_3.hr,<br>WY.14643_Low_24.hr,<br>WY.14643_Middle_9.hr,<br>WY.14643_Low_6.hr,<br>WY.14643_High_9.hr,<br>WY.14643_Middle_3.hr,<br>WY.14643_High_3.hr,<br>WY.14643_Low_9.hr,<br>WY.14643_High_24.hr,	aspirin_High_3.hr,<br>aspirin_Low_3.hr,<br>aspirin_Middle_9.hr,<br>aspirin_Low_6.hr,<br>aspirin_Middle_3.hr,<br>aspirin_High_6.hr,<br>aspirin_Middle_6.hr,<br>benzbromarone_Low_3.hr,<br>benzbromarone_Low_6.hr,<br>benzbromarone_Low_9.hr,<br>benzbromarone_Low_24.hr,<br>benzbromarone_Middle_24.hr,<br>cisplatin_Middle_6.hr,<br>cisplatin_Low_24.hr,<br>cisplatin_High_3.hr,<br>cisplatin_Middle_24.hr,<br>cisplatin_Middle_3.hr,<br>cisplatin_High_6.hr,<br>cisplatin_Low_6.hr,<br>cisplatin_Low_3.hr,<br>cisplatin_High_9.hr,<br>cisplatin_Low_9.hr,<br>cisplatin_High_24.hr,<br>cisplatin_Middle_9.hr,<br>clofibrate_Middle_3.hr,<br>clofibrate_Middle_24.hr,<br>clofibrate_High_3.hr,<br>clofibrate_Low_6.hr,<br>clofibrate_Low_3.hr,<br>clofibrate_Low_9.hr,<br>clofibrate_Low_24.hr,<br>diltiazem_High_6.hr,<br>diltiazem_Middle_3.hr,<br>diltiazem_High_24.hr,	

Cluster-1	Cluster-2	Cluster-3
	diltiazem_High_3.hr,<br>diltiazem_High_9.hr,<br>diltiazem_Middle_6.hr,<br>diltiazem_Low_9.hr,<br>diltiazem_Low_6.hr,<br>diltiazem_Low_24.hr,<br>diltiazem_Middle_24.hr,<br>diltiazem_Low_3.hr,<br>diltiazem_Middle_9.hr,<br>gemfibrozil_Low_9.hr,<br>gemfibrozil_Middle_6.hr,<br>gemfibrozil_Low_24.hr,<br>gemfibrozil_Low_3.hr,<br>gemfibrozil_Low_6.hr,<br>gemfibrozil_Middle_9.hr,<br>gemfibrozil_High_3.hr,<br>gemfibrozil_Middle_3.hr,<br>gemfibrozil_High_6.hr,<br>methapyrilene_Middle_24.hr,<br>methapyrilene_Low_9.hr,<br>methapyrilene_High_6.hr,<br>methapyrilene_Middle_3.hr,<br>methapyrilene_High_24.hr,<br>methapyrilene_High_3.hr,<br>methapyrilene_Low_3.hr,<br>methapyrilene_Low_6.hr,<br>methapyrilene_High_9.hr,<br>methapyrilene_Middle_9.hr,<br>methapyrilene_Low_24.hr,<br>methapyrilene_Middle_6.hr,<br>phenobarbital_Middle_3.hr,<br>phenobarbital_Low_24.hr,<br>phenobarbital_High_9.hr,<br>phenobarbital_High_24.hr,<br>phenobarbital_Middle_24.hr,<br>phenobarbital_Middle_9.hr,<br>phenobarbital_Low_9.hr,<br>phenobarbital_Middle_6.hr,<br>phenobarbital_Low_3.hr,<br>phenobarbital_Low_6.hr,<br>phenobarbital_High_6.hr,<br>phenobarbital_High_3.hr,<br>triazolam_High_24.hr,<br>triazolam_High_9.hr,<br>triazolam_Low_6.hr,<br>triazolam_Middle_6.hr,<br>triazolam_Middle_9.hr,<br>triazolam_Low_24.hr,<br>triazolam_Low_3.hr,<br>triazolam_High_3.hr,<br>triazolam_Middle_24.hr,<br>triazolam_High_6.hr,<br>triazolam_Middle_3.hr,<br>triazolam_Low_9.hr,	
